# Enhancing Child Digital Dietary Self-Monitoring via Positive Reinforcement: Proof-of-Concept Trial

**DOI:** 10.3390/nu17213341

**Published:** 2025-10-24

**Authors:** Lauren G. León, Elizabeth Anderson Steeves, Jeffrey Reinbolt, Tami H. Wyatt, Hollie Raynor

**Affiliations:** 1 Department of Nutrition, University of Tennessee, Knoxville, TN 37996, USA; lgriff31@vols.utk.edu; 2 Center for Nutrition and Health Impact, Omaha, NE 68154, USA; easteeves@centerfornutrition.org; 3 Department of Mechanical, Aerospace, and Biomedical Engineering, University of Tennessee, Knoxville, TN 37996, USA; reinbolt@utk.edu; 4 College of Nursing, University of Tennessee, Knoxville, TN 37996, USA; twyatt@utk.edu

**Keywords:** dietary self-monitoring, positive reinforcement, children

## Abstract

**Background/Objectives:** Dietary self-monitoring (DSM) is an essential behavior change strategy in pediatric nutrition interventions, but engagement is poor. Positive reinforcement (PR) techniques are used to improve behaviors in children. This proof-of-concept trial examined the ability to implement two types of PR, caregiver praise and gamification, using a digital DSM log. **Methods**: Families were recruited between February and October 2024. Children aged 8–12 years (*n* = 19) and an adult caregiver were randomly assigned to DSM conditions with or without caregiver praise and/or gamification (2 × 2 factorial design). Children tracked intake of fruits, vegetables, sweet/salty snack foods, and sugar-sweetened beverages for 4 weeks in a digital log. Feasibility focused on examining the amount of PR delivered. DSM behaviors (frequency and timing), child intrinsic motivation, and log usability and acceptability were also examined. **Results**: Gamification was implemented significantly more than caregiver praise. Caregiver praise was delivered on 12.2 ± 5.8 of 28 days, whereas gamification was delivered on 20.8 ± 12.3 of 28 days. There were no differences in DSM behaviors between PR conditions. Overall, children tracked on 23.6 ± 4.6 of 28 days, recorded 69.3% ± 45.1% of items on day of intake, and completed 23.1 ± 8.2 logging sessions. Additionally, there were no differences in child intrinsic motivation between PR conditions, and children and caregivers generally found the log usable and acceptable. **Conclusions**: The automation of gamification, which provides immediate, consistent, and convenient PR, may provide unique advantages for reinforcing child behaviors compared to praise, which relies on caregiver implementation.

## 1. Introduction

Dietary self-monitoring (DSM) is an essential behavior change strategy and is a cornerstone of nutrition interventions for children [1,2,3,4,5,6,7]. More complete DSM increases individuals’ awareness of their eating behaviors in relation to their dietary goals in real time, allowing them to make connections between their eating behaviors and health [8,9]. Additionally, the act of self-monitoring frequently produces positive changes in the tracked behavior, a phenomenon known as reactivity [10]. The tendency toward reactivity during self-monitoring makes it a beneficial behavioral change tool [11]. These characteristics of increased awareness and reactivity are hypothesized to improve nutrition choices and enhance health outcomes [4,5,6,7]. However, previous studies indicate that DSM adherence is poor among children and adolescents, especially over time [4,5,6,7].

Traditionally, DSM has been completed using pen-and-paper methods [12,13]. However, recent mobile technologies provide numerous options for digital diet tracking [14]. Smartphone applications (or apps) allow DSM that can be completed quickly, in real-time, while potentially reducing burden [12,13]. Examining how digital DSM can be enhanced to improve engagement in children is a needed area of research.

Because adherence to DSM is poor, strategies are needed to reinforce the behavior. Positive reinforcement (PR) is one such strategy for improving DSM. PR occurs when a reward is presented after a desired behavior is performed to increase the likelihood of that behavior occurring again [15]. Two common types of PR used within child-focused interventions are social reinforcers and token reinforcers [16,17]. Social reinforcement occurs when social rewards are provided following a behavior. For example, a child may receive a kind smile from a parent after engaging in positive behavior. Token reinforcement occurs when the reinforcer can be exchanged for something of value. For example, a child may receive points for completing chores at home, and these points may be exchanged for a reward.

A frequently targeted social reinforcer is caregiver praise. Praise signifies social acceptance, and social acceptance is reinforcing [18]. Caregiver praise is a fundamental component of parent training programs and is a frequently employed behavioral strategy in family-based treatment programs [18,19,20,21,22,23]. Furthermore, caregiver praise may be important for maintenance of outcomes after completion of family-based behavioral treatment programs [24]. Thus, the utility of caregiver praise for enhancing DSM behaviors specifically should be explored.

Gamification has been defined as “use of game design elements in non-game contexts” [25,26,27,28,29]. Digital points can be exchanged for rewards, such as badges, or “leveling up,” and therefore can be conceptualized as a form of token reinforcement. Blood glucose monitoring is one of the most explored areas in which the gamification of self-monitoring systems for children is used. Some, but not all, studies that utilized gamified blood glucose monitoring systems among children and adolescents with diabetes found evidence of improved self-monitoring [30,31,32]. Given these promising findings, research on how gamification can be integrated into DSM to enhance child outcomes should also be explored.

The unique qualities of caregiver praise and gamification as PR techniques may provide distinct advantages to each. PR is most effective when it is immediate, consistent, and convenient to deliver [19,33]. Digital gamification, which is automated, can deliver PR consistently and immediately. Additionally, given the ubiquity of digital devices in daily life, gamification can also be convenient to provide. Caregivers, on the other hand, may struggle to provide praise as quickly or regularly as automation given competing demands on attention. Furthermore, the extent to which a child engages in a behavior such as DSM may be explained by motivational factors. Self-determination theory provides a framework for understanding the relationship between PR and motivation [34]. Intrinsic motivation (i.e., when an individual engages in a behavior because of genuine interest, personal curiosity, and a desire for personal growth) is believed to support long-term maintenance of behaviors over time. Praise is generally believed to be beneficial for building a child’s intrinsic motivation [35,36], while research on how gamification affects intrinsic motivation is mixed [37,38]. Emerging evidence, however, suggests that when gamification is provided in the right way within the right context, it too can meet basic psychological needs and improve intrinsic motivation [37,38].

At this time, no studies have focused on how to improve the important behavioral strategy of DSM in children. Furthermore, there is a scarcity of research examining how PR, such as caregiver praise and gamification, could affect child DSM adherence. This proof-of-concept trial aimed to examine the feasibility of implementing two types of PR strategies (caregiver praise and gamification) within a digital DSM log. Secondary outcomes explored DSM behaviors (frequency and timing), child intrinsic motivation, and the usability and acceptability of the digital log.

## 2. Materials and Methods

### 2.1. Study Design and Primary Outcomes

This study was conducted in two phases: an initial formative testing phase and a subsequent proof-of-concept trial. This paper focuses on the proof-of-concept trial, but materials, methods, and results for formative testing are provided in detail in Supplementary File S1. Briefly, initial usability and acceptability of the DSM log was examined among five children aged 8–12 years and an adult caregiver. Participants completed a single in-person session to provide feedback on a beta version of the log. A concurrent think-aloud procedure was used, and transcripts were created. Transcripts were independently coded by two reviewers to identify recurring themes. If a majority (3 of 5) of children or caregivers mentioned the same issue, changes to the DSM log were made to address it prior to the proof-of-concept trial.

For the proof-of-concept trial, the DSM log was a mobile-optimized website, rather than an app, so that device operating system would not be a limitation of use. Families were therefore able to access the DSM log from a computer, smartphone, or other internet-enabled device.

Children were instructed to self-monitor their daily intake of the following food groups: fruits, vegetables, sweet and salty snack foods, and sugar-sweetened beverages (SSBs) for 4 weeks. DSM focused on these food groups because they have an established influence on health, are frequently targeted in childhood obesity treatment and prevention programs, and are easily understood by young children [23,39].

Using a 2 × 2 factorial design, child-caregiver dyads were randomly assigned to 1 of 4 conditions that varied in type of PR implemented: BASIC (no PR), PRAISE, GAME, or PRAISE + GAME. While DSM is frequently implemented within treatment, children in the proposed proof-of-concept trial engaged in DSM without a concurrent intervention to tightly control implementation of PR and measure its influence on DSM behaviors only (as compared to having all adult caregivers learning how to praise or having caregivers focus their praise on achieving dietary goals, which are both standard components of family-based, childhood obesity interventions).

To assess the feasibility of implementing PR strategies via a digital DSM log, this study examined the amount of PR (caregiver praise and gamification) delivered to children. Additionally, DSM frequency (i.e., number of days any food/beverage item was tracked and/or logging was marked complete), DSM timing (i.e., the proportion of foods logged on the day of intake and the number of logging sessions each day), changes in child intrinsic motivation, and usability and acceptability of the DSM log were also examined.

### 2.2. Participants

**Figure 1:**
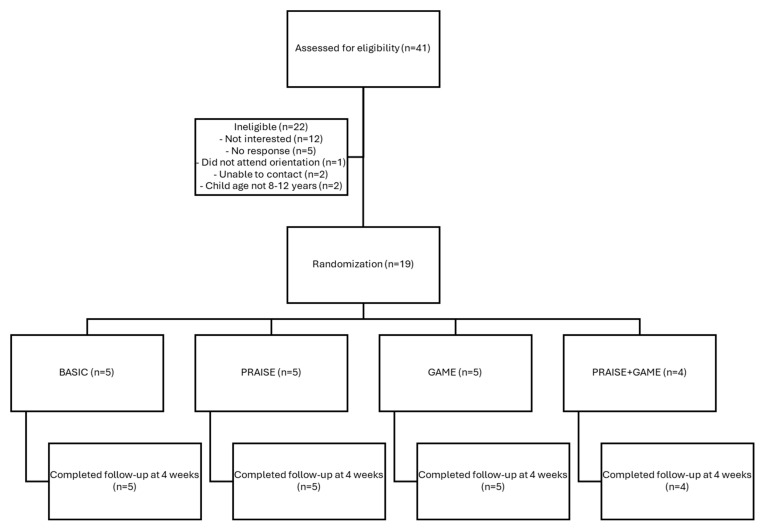
Participant flowchart.

Figure 1 displays the study flow diagram outlining enrollment, randomization, and follow-up of participants. Children ages 8–12 years at or above a healthy weight [defined as body mass index (BMI)-for-age ≥ 5th percentile] who reported consuming foods/beverages from ≥2 targeted food groups (i.e., fruits, vegetables, sweet and salty snack foods, and sugar-sweetened beverages) on ≥3 days/week each and who had an adult caregiver ≥18 years of age willing to participate were eligible for enrollment. Families were excluded if the child was already self-monitoring their diet or had major psychiatric diseases or organic brain syndromes, or if the family did not live in the greater Knoxville area or speak English. Additionally, families were required to have reliable access to the internet via phone, computer, or another device that the child was able to operate.

Recruitment occurred from February through October of 2024. Recruitment strategies included internal campus mailings to faculty and staff across the university campus, as well as in-person tabling at community events and after-school programs. Additionally, recruitment flyers were posted in public spaces in the Knoxville area (e.g., libraries, toy stores), and the study was advertised on social media platforms. All study advertisements included a QR code (paper flyers) or link (social media ads) to a Qualtrics form (Qualtrics, Provo, UT, USA) that collected contact information and obtained permission to contact. At the end of the 4-week DSM period, families who completed follow-up assessments received two $25 gift cards, one for the child and one for the caregiver, and were provided access to a short online behavioral nutrition education program (additional details in Section 3.4).

As this was a proof-of-concept trial, targeted enrollment was 40 families (10 families per group). Previous research suggests that small sample sizes are appropriate for initial testing of novel health technologies [40,41,42].

### 2.3. Digital Dietary Self-Monitoring Log

**Figure 2:**
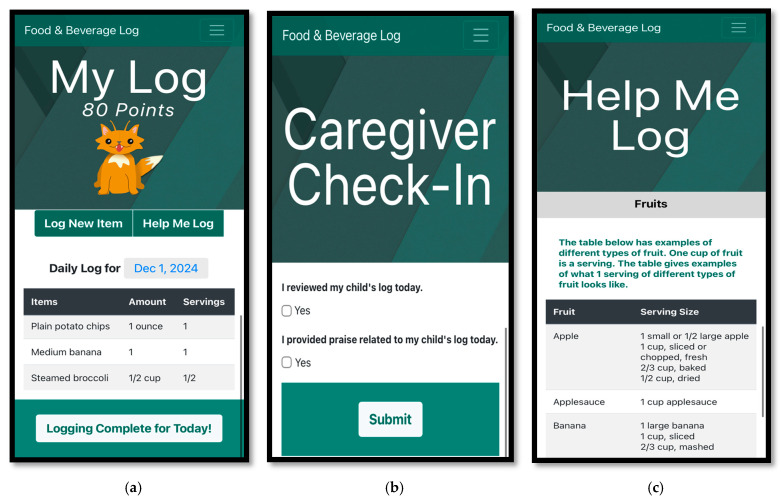
Example images of the dietary self-monitoring log as viewed from a mobile device. (**a**) An example of a child’s DSM log with gamification. At the top, points are displayed above the virtual pet. Below the pet, options to log an item or access the help feature are available. The date is shown for the displayed day of logging and can be changed to view alternative days. At the bottom, a button to mark logging complete for the day is included. (**b**) An example of the caregiver check-in with praise check-in (only accessible from the caregiver URL). (**c**) An example of the fruits group help page within the help feature.

Figure 2 provides example images of the dietary self-monitoring log as viewed from a mobile device.

#### 2.3.1. BASIC

Child DSM logs were accessed via a unique URL. The BASIC DSM log included four features: (1) the ability to log a food or beverage with amount and servings, (2) the ability to mark logging complete for the day, (3) a help feature with information on servings sizes for the targeted food groups, and (4) a check-in that allowed caregivers to indicate they had reviewed their child’s log that day. Caregivers were provided with a separate URL that allowed them to view their child’s logging and complete check-ins. Entries submitted in the DSM logs automatically populated a web-based spreadsheet that collected participant identification number, date and time of entry, food/beverage name, amount, serving size, and caregiver check-ins.

At the in-person appointment, families completed a DSM practice session under observation of research staff using the family’s personal DSM. The family practiced logging foods and beverages from a standard list and were then instructed to mark logging as complete for the day. Research staff answered any questions the family had and explained how to use the “Help Me Log” feature (Figure 2c). Caregivers were also shown how to complete the caregiver check-in feature (Figure 2b). Additionally, caregivers were encouraged to avoid negative reinforcement (e.g., nagging). If the caregiver noticed the child not using the DSM log, they were encouraged to engage in problem solving and preplanning, rather than nagging, to support their child. If the caregiver noticed the child engaged in undesirable behaviors (e.g., recording unhealthy foods), they were encouraged to ignore rather than criticize.

To help children get started, caregivers were instructed to sit with their child for the first 3 days of tracking to help with logging. Caregivers were also encouraged to help their child with logging activities whenever necessary throughout the 4-week DSM period.

#### 2.3.2. PRAISE

In addition to the procedures for the BASIC log, caregivers of families assigned to PRAISE were asked to provide process praise, a type of praise that emphasizes effort over outcomes or character traits, to their child for engaging in DSM over the 4 weeks. This type of praise has been shown to support intrinsic motivation in children [43]. As there were no intervention goals, caregivers were instructed to only praise one behavior—DSM. Additionally, when caregivers in this group completed check-ins, an additional item was present to indicate whether praise was provided the same day (Figure 2b).

During the in-person appointment, caregivers also provided process praise to their child during the DSM practice session so that research staff could observe and provide feedback. A description of process praise was provided, including a list of examples, and caregivers completed a brief “quiz” in which they attempted to identify process praise statements. Additionally, caregivers were encouraged to provide praise as close to the occurrence of the behavior as possible to increase its effectiveness. Completion of the caregiver check-ins included an additional item related to whether praise had been provided (Figure 2b).

#### 2.3.3. GAME

In addition to the procedures described for BASIC, the logs of children in GAME integrated three game mechanics: points, levels, and a virtual pet. Points were earned for logging food or drink, marking logging complete for the day, and logging two days in a row. The virtual pet had 12 levels of evolution (Supplementary Figure S1), with each level requiring more points to achieve than the previous level (e.g., 2 points for level 2, 4 points for level 3, 8 points for level 4, etc.). The twelfth and final level was achieved after accruing 111 points. Virtual pets have previously shown the potential to improve weight-related behaviors in children in other contexts [44,45,46].

During the in-person appointment, children and caregivers observed how points were accrued in the family’s personal log during the DSM practice session. Prior to the practice session, research staff explained how points were earned and how points helped to “level up” the virtual pet (Figure 2a). Points were accrued and represented in the DSM log in real time so that children were immediately rewarded for engaging in DSM behaviors.

#### 2.3.4. PRAISE + GAME

For families assigned to PRAISE + GAME, procedures included all of those described above for BASIC, PRAISE, and GAME.

### 2.4. Flow of Sessions

After an initial phone screening to determine eligibility, interested families were invited to attend an in-person orientation to learn more about the study. For families who agreed to participate, caregivers provided informed consent, children provided assent, and baseline assessment measures were collected (see Section 2.5).

Participants were randomly assigned to one of four conditions (BASIC, PRAISE, GAME, or PRAISE + GAME) using a pre-generated randomization list. The list was created by the principal investigator using a random number generator to produce sets of four unique integers (1–4), each corresponding to one of the four conditions. As families enrolled in the study, they were assigned to the next available condition on the list in order of enrollment. As research staff were required to provide families with detailed instructions regarding praise delivery and/or the mechanics of the gamified system, allocation concealment was not possible. Both staff and participants were aware of group assignments.

Immediately after baseline measures were completed, families were told their assigned condition and received brief training on using the log. All families received information on the benefits of DSM and the relationships of the four targeted food groups with child health. Families were also informed that the child’s DSM records over the next 4 weeks would be used to provide recommendations to the family at their follow-up visit, at which time they would receive access to a short online behavioral nutrition education program (described below).

Each family was provided with access to a log that matched group assignment, and children were asked to complete DSM for 4 weeks. Given previous studies had reported low adherence to DSM logs within one month, 4 weeks were expected to be appropriate for detecting differences in DSM behavior by group. The DSM period began the day after the family’s baseline assessment.

Caregivers were instructed to review their child’s DSM and complete a caregiver check-in each day. Additionally, to get children started, all caregivers were instructed to sit with their child each day for the first 3 days of the DSM period to help their child log any items consumed from targeted food groups.

At the end of the 4-week DSM period, families attended a virtual appointment to complete follow-up measures. To provide feedback to families, tracking from days on which tracking was marked as complete was entered into the Nutrition Data System for Research (NDSR, version 2022; Nutrition Coordinating Center [NCC], University of Minnesota, Minneapolis, MN, USA) software to determine servings of fruits and vegetables consumed and intake of added sugars and saturated fats from sugar-sweetened beverages and sweet and salty snack foods. These values were entered into a template feedback sheet that compared the child’s intake to the recommendations in the Dietary Guidelines for Americans 2020–2025. Families were informed that dietary intake extracted from the child’s DSM log provided a general estimate of what the child may be consuming and that very incomplete or incorrect diaries would reduce accuracy of feedback.

Families were also provided with access to a short online behavioral nutrition education program that consisted of four video modules with the following topics: (1) dietary guidelines recommendations and basic nutrition information, (2) establishing a healthy home environment, (3) PR of healthy behaviors, and (4) pre-planning and problem solving.

### 2.5. Measures

#### 2.5.1. Sample Characteristics (Baseline Only)

Demographics. Demographic information included child and caregiver age, race, ethnicity and gender; caregiver marital status, education level, and employment status; and household income.

Child height and weight. Child height and weight were collected to confirm study eligibility, and body mass index (BMI) z-score was calculated based on sex and age. Height and weight measures were collected by trained research staff in the laboratory setting.

#### 2.5.2. Primary Measures: Adherence and PR Dose (During 4-Week DSM Period)

Caregiver check-ins. To determine the extent to which caregivers adhered to instructions to review their child’s log each day, the number of days on which caregivers marked “yes” for the item “I reviewed my child’s log today” was recorded.

Caregiver praise. To examine whether the caregiver praise manipulation was successfully implemented, the number of days on which caregivers marked “yes” for the item “I provided praise related to my child’s log today” was recorded.

Gamification. To examine whether the gamification manipulation was successfully implemented, the number of days on which children received ≥1 point was calculated.

#### 2.5.3. Secondary Measures: DSM Behaviors, Child Intrinsic Motivation, and Usability and Acceptability of the Digital Log

DSM frequency (during 4-week DSM period). Frequency was operationalized as the number of days with any tracking. Absence/presence of DSM was coded as ‘0’ or ‘1’ for each day, with ‘0’ representing no food or beverage tracked AND tracking not marked as complete for the day and ‘1’ representing ≥1 food or beverage tracked AND/OR tracking marked as complete for the day. In other words, a day was counted as “tracked” if any food or beverage was logged on that day or, if no food or beverage is logged, the “Logging Complete” button was clicked. The possible range for overall DSM frequency for the 4-week period ranged from 0 (no DSM any day) to 28 (DSM on all days). The possible range for DSM frequency by week ranged from 0 to 7.

DSM timing (during 4-week DSM period). Timing was operationalized two ways: (1) the mean number of DSM logging sessions per day (must be >15 min apart to be considered distinct sessions) and (2) the proportion of food or beverage items tracked on the day of intake (i.e., the number of food or beverage items tracked on the day of intake divided by the total number of food or beverage items tracked). Thus, the mean number of DSM logging sessions per day was a continuous variable, and the possible range for the proportion of items tracked on day of intake ranged from 0 to 1.

Child intrinsic motivation (baseline and follow-up). The Task Evaluation Questionnaire of the Intrinsic Motivation Inventory (IMI) was administered to determine whether differences in pre-post changes in child intrinsic motivation were present [47,48]. The questionnaire consists of 22 items and uses a 5-point Likert scale (not at all true to very true) to assess four subdomains: interest/enjoyment, perceived choice, perceived competence, and pressure/tension. Though originally developed for use in adult populations [49], the IMI has also been used in numerous research areas with child participants, including first language, mathematics, sport activities, and learning, and has been shown to be a valid measure in children 9 years and older [50,51,52,53].

Child/Caregiver usability and acceptability survey (follow-up only). The survey used in the formative phase was also administered at follow-up to capture families’ experiences with the DSM log. The survey was developed based on the usability and acceptability survey items described by Marsac et al. in their feasibility testing of a web-based application that included gamification and targeted children between ages 8–12 years [54]. Though not validated, the original survey provided a framework for adapting simple, straightforward questions related to usability and acceptability.

### 2.6. Statistical Analysis

Data were analyzed using SAS Enterprise Guide 8.3 (SAS Institute Inc., Cary, NC, USA). Summary statistics were used to describe sample characteristics. For the full sample, continuous variables were summarized as mean ± standard deviation if normally distributed and as median (interquartile range) if not. Normality was assessed using the Shapiro–Wilk test. Visual inspection of distributions was used to complement formal testing where appropriate. Formal normality testing within randomized groups was not performed due to small sample sizes, as Shapiro–Wilk has limited power in very small samples. Thus, medians and interquartile ranges are presented for all randomized group-level data.

Differences in baseline characteristics by group were assessed using appropriate statistical tests based on the type and distribution of the data. Categorical variables were compared using Fisher’s exact test to account for cell counts <5, and continuous variables were compared across groups using nonparametric Kruskal–Wallis tests, as small group-level sample sizes limited reliable assessment of normality.

Differences in the number of caregiver check-ins completed by assignment to PR type (caregiver praise or gamification) were examined using nonparametric Mann–Whitney U tests. Separate tests were conducted for caregiver praise and gamification, each with a dependent variable of number of completed caregiver check-ins.

To examine implementation of the PR techniques, a linear mixed-effects model was used with two within-subject factors of PR type (caregiver praise vs. gamification) and time (week), including their interaction, and a dependent variable of dose. Dose was defined as the mean number of days per week on which caregivers responded affirmatively to praise check-ins and the mean number of days on which ≥1 point was gained per week were calculated for families assigned to caregiver praise and gamification, respectively. Random intercepts accounted for repeated measures within participants.

To determine the main effects of PR and time on DSM behavior, linear mixed-factor ANOVA was used with between-subject factors of praise and gamification assignment, a within-subject factor of time (week), and a dependent variable of DSM behavior (frequency or timing). Random intercepts accounted for repeated measures within participants. Residuals demonstrated some skewness, suggesting departure from normality, but mixed models are generally robust to such violations [55]. Additional sensitivity analyses using a log-transformed outcome produced consistent results, supporting the robustness of the primary findings.

To determine the main effects of PR on motivation, a 2 × 2 factorial ANCOVA was conducted with gamification and caregiver praise assignment as the independent variables, a dependent variable of intrinsic motivation at follow-up, and a covariate of intrinsic motivation at baseline.

A standard alpha level of <0.05 was used to determine statistical significance. Tukey’s adjustment was applied for post hoc pairwise comparisons.

Effect sizes were calculated using the formula for partial omega-squared ($\omega_{p}^{2}=\frac{{SS}_{effect}-({df}_{effect}\times {MS}_{error})}{{SS}_{effect}+{SS}_{error}+{MS}_{error}})$. Omega-squared is a more conservative measure appropriate for studies with small sample sizes that utilize ANOVA [56], and partial omega-squared, in particular, is most appropriate for multi-factor designs because it examines the proportion of variance in the dependent variable that is uniquely attributed to each independent variable or interaction, while controlling for other factors in the model [57]. Thresholds for small, medium, and large effects were defined as 0.01, 0.06 and 0.14, respectively, as previously defined by Cohen [58].

## 3. Results

### 3.1. Child and Caregiver Demographics

In total, 19 caregiver–child dyads entered the trial. Group-level baseline characteristics are shown in Table 1. The children had a mean age of 9.6 ± 1.3 years, with 58% female, 5% identifying as Hispanic or Latino, and 74% identifying as white. Average child BMI percentile was 60.6 ± 30.8. Caregivers had a mean age of 42.8 ± 4.9 years; 79% were female, none reported being Hispanic or Latino, and 79% identified as white. Most caregivers were married (89%), had at least four years of college education (89%), and reported household incomes of $100,000 or more annually (84%).

### 3.2. Completion of Caregiver Check-Ins

Caregivers completed check-ins for a median of 15 (IQR: 6–16) days out of a possible 28 days. Caregiver check-in completion did not differ by condition. Results from Mann–Whitney U test showed no differences in the number of caregiver check-ins completed by assignment to caregiver praise (*S* = 85.5, *Z* = −0.33, *p* = 0.75) or gamification (*S* = 99.5, *Z* = −0.74, *p* = 0.23).

### 3.3. Implementation of PR

Caregivers assigned to a condition that contained praise indicated they provided praise (via praise check-in) for a median of 13 (IQR: 9–14) praise check-ins out of a possible 28 days. Children assigned to a condition that contained gamification received ≥1 point a median of 22 (IQR: 16–25) out of a possible 28 days and earned a median of 197 (IQR: 182–225) points during the DSM period. Out of the 9 children assigned to a condition that contained gamification, 7 achieved the maximum virtual pet level of 12, with the 2 remaining children achieving levels 10 and 11.

**Figure 3:**
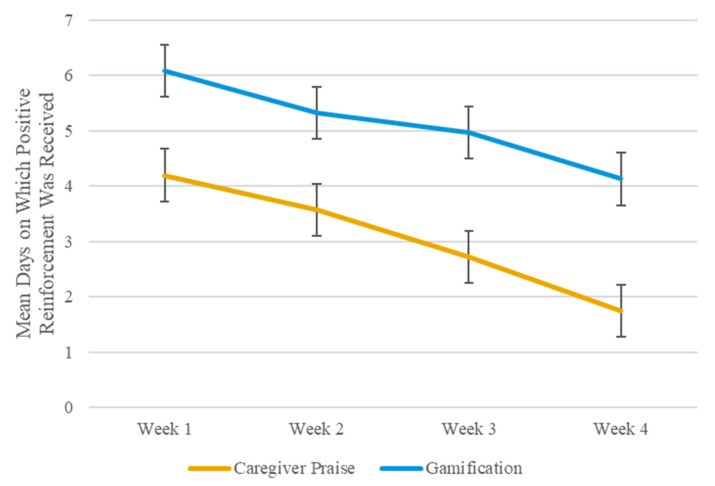
Mean days per week on which dose of caregiver praise (i.e., caregiver praise check in completed) or gamification (i.e., ≥1 point received) was received. Figure presents marginal means and standard error. Marginal means (calculated as Least Squares Means in SAS) and standard errors predicted by linear mixed-effects model testing are presented.

Children assigned to gamification appeared to receive significantly more reinforcement than those assigned to caregiver praise. Linear mixed-effects model testing showed a main effect of PR, such that receipt of gamification was significantly higher across the 4-week DSM compared to caregiver praise [median of 3.0 (IQR: 1.5–4.0) days per week versus 5.0 (IQR: 4.0–7.0) days per week, *F*(1,3) = 29.17, *p* = 0.01]. Additionally, there was a main effect of time [*F*(3,39) = 14.67, *p* < 0.0001], with dose of both caregiver praise and gamification decreasing across the DSM period. Figure 3 illustrates the median days per week on which caregiver praise and points were received by children assigned to these respective conditions.

### 3.4. Dietary Self-Monitoring Behaviors

Participants recorded food and beverage items for a median of 24.0 days (interquartile range: 21.0–27.0) of the 28-day study period, tracked 69.3% ± 45.1% of items on the day of intake, and completed 23.1 ± 8.2 logging sessions. PR assignment did not significantly affect DSM behaviors. Linear mixed-factor ANOVA testing revealed no significant main effects of PR on number of days tracked [overall model: *F*(3,15) = 1.75, *p* = 0.20], proportion of items tracked on the day of intake [overall model: *F*(3,15) = 0.42, *p* = 0.74], or number of logging sessions [overall model: *F*(3,15) = 0.27, *p* = 0.85]. Table 2 presents medians and IQRs for DSM measures by caregiver praise and gamification level.

Additionally, DSM behaviors decreased over time (Table 2). This suggests a decline in engagement over the 4-week period. Linear mixed factor ANOVA testing revealed a significant effect of time on average number of days with tracking [*F*(3,48) = 6.15, *p* = 0.001], proportion of items tracked on day of intake [*F*(3,43) = 4.03, *p* = 0.01], and number of logging sessions [*F*(3,48) = 31.7, *p* < 0.0001].

### 3.5. Intrinsic Motivation

Table 3 presents scores for intrinsic motivation subdomains. Gamification appeared to enhance interest/enjoyment but had no significant effects on other motivation subdomains. Only assignment to gamification had a significant main effect on the IMI subdomain of interest/enjoyment [*F*(1,15) = 4.66, *p* = 0.048], while the overall model was not significant [*F*(3,15) = 3.14, *p* = 0.06]. There were no other significant main effects of assignment to caregiver praise or gamification on the IMI subdomains of perceived competence [overall model: *F*(3,15) = 6.26, *p* = 0.004], perceived choice [overall model: *F*(3,15) = 4.10, *p* = 0.02], or pressure/tension [overall model: *F*(3,15) = 1.94, *p* = 0.16] after controlling for baseline scores.

### 3.6. Usability and Acceptability

Full results from the usability and acceptability survey are presented in Supplementary Table S1. In short, the majority of children (79%) and caregivers (53%) reported the log was “very easy to use.” Most children and caregivers reported it was “not at all confusing” to use the log (53% and 84%, respectively) or help feature (74% and 100%, respectively). Thirty-seven percent of children and caregivers each said the help feature “maybe” had too many words. Most caregivers (84%) indicated that the caregiver check-in feature was “not at all confusing.” Almost three-quarters (74%) of children indicated the log was “somewhat” or “a lot of fun” to use, and 68% of caregivers indicated their child enjoyed using the log “somewhat” or “a lot.” Eighty-four percent of children and caregivers each indicated they like the appearance of the log “somewhat” or “a lot.” A total of 47% of children and 37% of caregivers said they would recommend the log to other kids/families, and 42% of children and 37% of caregivers indicated that they would use the log again.

## 4. Discussion

### 4.1. Study Overview

This proof-of-concept trial examined the ability to implement two types of PR, caregiver praise and gamification, using a digital DSM log. Despite DSM being a fundamental component of pediatric nutrition intervention, no previous studies have focused on how to improve this important behavior or how PR could affect child DSM. In this study, implementation of PR differed significantly by type. Children assigned to gamification received a higher dose of PR in comparison to children assigned to caregiver praise across the 4-week DSM period. Additionally, children in the current study engaged in unexpectedly high levels of DSM, indicating high feasibility of implementation. Results from this proof-of-concept trial suggest that PR delivered via digital DSM logs is feasible and warrants further investigation in larger trials in the future.

### 4.2. Implementation of Caregiver Praise Versus Gamification

On average, children in this study received gamified PR on 9 more days than caregivers reported providing PR by praise. During week 1 of this study, children assigned to gamification received at least one point approximately every day, while caregivers assigned to praise completed praise check-ins on approximately 4 out of 7 days. By the final week of the DSM period, children assigned to gamification received at least one point on approximately 5 out of 7 days, while caregivers assigned to praise completed praise check-ins on fewer than 1 out of 7 days on average. Thus, gamification appeared to be implemented at much more ideal levels than caregiver praise.

The manner in which PR is implemented is strongly related to its success in establishing a behavior [19,33]. Three important factors are the consistency, immediacy, and convenience with which a PR strategy can be implemented [33]. These three qualities are characteristic of PR provided via gamification within a digital system. First, gamification is automated and is therefore delivered consistently after the behavior occurs. The automation also tightly pairs the reinforcement with the behavior, increasing the likelihood that the behavior will occur again. Finally, a gamified system experiences no inconvenience in implementing its PR mechanics, and gamification often fits conveniently into daily life given the ubiquity of mobile phones and other electronic devices [59]. In contrast, caregivers who have competing demands on their time and attention may struggle to praise child behavior in a manner as consistent or immediate as automation. While data on the immediacy of praise were not collected in the current study, the immediacy of caregiver praise is reliant on human behavior and depends on several factors: the caregiver must be present with the child at the time of the behavior, must notice the behavior has occurred, and subsequently must remember to provide the praise in a timely manner. Thus, it is likely that caregivers were unable to provide PR with the same immediacy as automated gamification. These factors may also mean that praise is at times inconvenient for caregivers to provide, which may lead to the opportunity for providing PR to be missed. Thus, while caregiver praise and gamification both may act as effective positive reinforcers, gamification potentially offers unique advantages. This may be particularly true over time as positive reinforcers that are reliant on human behavior (e.g., caregiver praise) may diminish (as demonstrated by the diminished provision of praise in the current study). Still, further research is needed to examine the effects of social reinforcement (e.g., praise), which is based around human interaction and connection, versus gamified reinforcement, which relies on digital automation, on child behaviors in the long term.

### 4.3. Patterns in DSM Engagement

Participants in the current investigation also engaged in unexpectedly high levels of DSM frequency, though the reason is unclear. One potential explanation is that families in this study were not enrolled in concurrent intervention. In the absence of other intervention goals, children did not have to split their focus between DSM and other behaviors. Additionally, children were only asked to self-monitor four food groups. Past studies reporting DSM outcomes in children implemented self-monitoring within the context of obesity treatment, which may have included more sophisticated tracking (e.g., calories) [4,5,6,7]. This method of DSM would necessarily be more time-consuming and burdensome and therefore may explain some of the reduced engagement compared to the current study.

Both DSM frequency and timing decreased significantly over the 4-week DSM period. However, participants engaged in unexpectedly high levels of DSM frequency compared to past studies in which half of children engaged in little to no DSM, at least during obesity treatment [5,6,7]. Additionally, about two-thirds of logged items were recorded on the day of intake, and participants completed slightly less than one logging session per day on average. Timing of DSM is important because reducing the time between intake and recording decreases the chance that errors will be made due to recall bias, a common issue encountered in dietary recall [60,61]. As no other studies have reported on DSM timing in children, it is unclear whether these outcomes are more optimal than expected.

Overall, this proof-of-concept trial suggests children and families are willing to engage with digital self-monitoring systems at high levels. The DSM log used in the current study is highly adaptable to other interventions and contexts as the caregiver praise and gamification system could easily be applied to self-monitoring of any targeted behavior. Taken together, these findings highlight the potential use of digital PR systems in wide-ranging applications across pediatric health behavior interventions. Researchers may also consider implementing other automated aspects into the digital tool, such as regular caregiver reminders via text messages to prompt frequent praise and encourage them to use effective praise statements.

### 4.4. Effects of Caregiver Praise on DSM Behaviors and Intrinsic Motivation

Children assigned to receive caregiver praise did not show significant differences in DSM frequency, timing, or intrinsic motivation compared to those who did not. Praise has long been recognized as a valuable reinforcement strategy [19,62], and caregivers were trained to use process praise daily. However, praise check-ins occurred on fewer than half of days and decreased over time—suggesting insufficient dose. Thus, lack of consistency may have reduced the effectiveness of praise to influence outcomes. Additionally, it was unclear how praise was implemented in the real world. If caregivers unintentionally used a less desired type of praise (e.g., person or product praise), or if the praise was perceived as insincere or controlling, it may have reduced its effectiveness [43,63,64,65].

### 4.5. Effects of Gamification on DSM Behaviors and Intrinsic Motivation

Gamification also had no significant effect on DSM frequency or timing. However, this finding should be interpreted cautiously given the nonsignificant overall test. While prior research on gamified DSM is limited, some studies have found benefits to related behaviors such as blood glucose monitoring or dietary intake [30,31,32,66,67]. In this study, gamification dose was higher than praise, and though non-significant, it had a medium effect size on DSM frequency.

Children assigned to gamification did report higher levels of intrinsic motivation on the interest/enjoyment subdomain compared to children who were not assigned to gamification. Participants assigned to gamification tracked on average about 3 more days than controls. The consistency, immediacy, and convenience of automated PR may have contributed to this finding. Research on how gamification affects intrinsic motivation is mixed [37,38,68,69], but emerging evidence suggests that when gamification is provided in the right way within the right context, it too can meet basic psychological needs and improve intrinsic motivation [37,38]. There is some evidence that virtual agents, such as pets, may increase feelings of relatedness [70,71].

Additionally, post hoc power analysis was conducted using G*Power (version 3.1.9.7; Heinrich Heine University, Düsseldorf, Germany [72]) to calculate the achieved statistical power. The effect size of gamification on frequency of DSM ($\omega_{p}^{2}=0.09$) was used as it demonstrated the largest observed effect of PR type on DSM behaviors. Analysis was based on a factorial ANOVA with two groups (assigned to gamification vs. not assigned to gamification), an alpha level of 0.05, and a total sample size of 19. Achieved power for correctly detecting an effect of assignment to gamification on DSM frequency was calculated to be 42%, indicating that the study was substantially underpowered. This limitation highlights the need for future studies with larger samples to more reliably detect potential effects.

### 4.6. Perceptions of Usability and Acceptability

In general, the log was well received in the proof-of-concept trial. Participants felt the log was easy to use and did not report high levels of confusion. Nearly three quarters of children indicated that the log was at least somewhat fun to use, and about two thirds of caregivers felt their child at least somewhat enjoyed using the log.

Despite this positive feedback, less than half of children and caregivers indicated that they would be willing to use the log again or would recommend it to others. However, this may be attributed to the fact that the log was used without any concurrent intervention or dietary goals. Thus, families may not have felt that using the log to track food and beverages in isolation of a broader context was valuable.

A notable theme identified in both child and caregiver feedback during formative testing was concern about the potential confusion children might feel when trying to log food or beverages with serving sizes and amounts. To address this, structured training on how to use the logging feature was provided during the training session at proof-of-concept trial baseline visits. Still, about half of the children indicated that logging food and drinks was somewhat to very confusing at the end of the trial, suggesting that tracking in this manner is a difficult skill to master for children aged 8–12 years. It is only beginning around the age of 8 years that children’s ability to self-report dietary intake rapidly increases, which means that DSM may still be challenging for children at the lower end of the enrolled age range [73]. Thus, younger children may require increased assistance from an adult or caregiver to engage in DSM at optimal levels.

### 4.7. Strengths and Limitations

Strengths of the study include the decision to compare caregiver praise and gamification, which allowed for examination of two PR strategies with differing characteristics. The digital gamification system also allowed for full, accurate capture of dose of PR received via gamification. Additionally, the use of a web-based DSM log allowed for the automated recording of timestamps that could be compared to the day on which the item was reported as consumed, and this was also the first study to our knowledge that examined the timing of DSM behaviors in children and adolescents. Furthermore, the 2 × 2 factorial design allowed for the isolation of the effects and interactions of PR on DSM, and a potential underlying mechanism (intrinsic motivation) that might explain why PR affects DSM was also explored.

The primary limitations of the study include implementation of caregiver praise and the method for capturing whether praise was delivered. Caregivers completed praise check-ins on less than half of the intended days during the proof-of-concept trial, indicating that they may have encountered barriers to providing daily process praise. However, it remains unclear whether the challenges were related to providing praise or simply completing the check-in in a timely manner. Furthermore, caregivers were not required to record the praise statements they provided to their children, which prohibited examination of whether caregivers were appropriately providing process praise statements according to study procedures. Additionally, while the virtual pet system implemented in this study appeared to work well and be well-received, there are many alternative gamification mechanics (e.g., leaderboards, customization) that could have been tested to determine their influence on sustaining child engagement in DSM [25,26,27,28,29]. Finally, the small sample size and the homogeneity of participants restricted statistical power and limited generalizability. Recruitment was discontinued prior to reaching the planned sample size due to time constraints and low response rates among eligible families. These factors limited the feasibility of continued enrollment despite initial targets. Additionally, the sample was not diverse and included predominantly white children from high income families. This may have influenced both adherence to the intervention and perceptions of the usability and acceptability of the digital log. These demographic characteristics may have contributed to the relatively high levels of engagement observed and should be considered when interpreting the findings. As such, the results may not generalize to more diverse populations or families with fewer resources or less education, and future research should aim to recruit more representative samples to better understand the broader applicability of digital PR systems.

## 5. Conclusions

This proof-of-concept trial aimed to examine the feasibility of implementing PR within a digital DSM log. Overall, this study suggests that children and their families are willing to engage in DSM at high levels when using a web-based DSM log. In fact, children in this study engaged in DSM levels much higher than expected based on the past research. Additionally, integrating PR into the DSM process appears feasible, and neither caregiver praise nor gamification appears to negatively impact intrinsic motivation in children. Results suggest automated PR, such as gamification, can be implemented successfully with high levels of fidelity. The automation may provide additional advantages as a PR strategy, as it ensures reinforcement of behavior is offered immediately, consistently, and conveniently without relying on human behavior.

## Figures and Tables

**Table 1:** Proof-of-concept trial sample characteristics by group.

Variable	BASIC (n = 5)	PRAISE (n = 5)	GAME (n = 5)	PRAISE + GAME (n = 4)	*p*-Value
Child age, Mdn (IQR)	9.5 (8.8–9.8)	9.6 (9.2–9.7)	11.1 (11.0–12.1)	9.4 (8.9–10.8)	0.2
Child sex, n (%) ^1^					
Female	4 (80%)	2 (40%)	3 (60%)	2 (50%)	0.8
Male	1 (20%)	3 (60%)	2 (40%)	2 (50%)	
Child ethnicity, n (%)					
Hispanic or Latino	1 (20%)	0 (0%)	0 (0%)	0 (0%)	1.0
Not Hispanic or Latino	4 (80%)	5 (100%)	5 (100%)	4 (100%)	
Child race, n (%)					
White	4 (80%)	3 (60%)	5 (100%)	2 (40%)	
Black or African American	0 (0%)	0 (0%)	0 (0%)	1 (20%)	0.7
Asian	0 (0%)	1 (20%)	0 (0%)	0 (0%)	
Other or more than one race	1 (20%)	1 (20%)	0 (0%)	1 (20%)	
Child BMI %ile, Mdn (IQR)	30.0 (28.0–35.0)	95.0 (66.0–97.0)	91.0 (42.0–92.0)	66.0 (40.5–79.5)	0.3
Caregiver age, Mdn (IQR)	43.0 (41.0–44.0)	41.0 (40.0–45.0)	44.0 (42.0–46.0)	42.5 (36.0–46.0)	0.7
Caregiver sex, n (%) ^1^					
Female	3 (60%)	3 (60%)	5 (100%)	4 (100%)	0.3
Male	2 (40%)	2 (40%)	0 (0%)	0 (0%)	
Caregiver ethnicity, n (%)					
Not Hispanic or Latino	19 (100%)	19 (100%)	19 (100%)	19 (100%)	nd
Caregiver race, n (%)					
White	5 (100%)	3 (60%)	5 (100%)	2 (40%)	
Black or African American	0 (0%)	0 (0%)	0 (0%)	1 (20%)	
Asian	0 (0%)	1 (20%)	0 (0%)	0 (0%)	0.2
Other or more than one race	0 (0%)	1 (20%)	0 (0%)	1 (20%)	
Caregiver marital status, n (%)					
Married	5 (100%)	5 (100%)	4 (80%)	3 (75%)	
Never married	0 (0%)	0 (0%)	1 (20%)	0 (0%)	0.6
Refused to answer	0 (0%)	0 (0%)	0 (0%)	1 (25%)	
Caregiver education level, n (%)					
Grade 12 or GED (high school graduate) or less	0 (0%)	1 (20%)	0 (0%)	0 (0%)	1.0
College 1 to 3 years (some college or technical school)	0 (0%)	0 (0%)	1 (20%)	0 (0%)	
College 4 years or more (college graduate)	5 (100%)	4 (80%)	4 (80%)	4 (100%)	
Household income, n (%)					
Less than $59,999	0 (0%)	0 (0%)	1 (20%)	0 (0%)	
$60,000 to $79,999	0 (0%)	1 (20%)	0 (0%)	1 (25%)	0.3
$100,000 or more	5 (100%)	4 (80%)	4 (80%)	3 (75%)	

^1^ All participants reported the matching responses for sex and gender. Abbreviations: BMI = body mass index; Mdn (IQR) = median (interquartile range); nd = no difference between groups.

**Table 2:** Dietary self-monitoring outcomes by PR status for the overall period.

Measure	Caregiver Praise			Gamification			Time ^2^				
	No (n = 10)	Yes (n = 9)	Main Effect	No (n = 10)	Yes (n = 9)	Main Effect	Week 1	Week 2	Week 3	Week 4	Main Effect
Number of days with tracking, Mdn (IQR) (range: 0 to 28)	24.0 (21.0, 27.0)	26.0 (21.0, 27.0)	*p* = 0.5	22.5 (20.0, 27.0)	26.0 (23.0, 28.0)	*p* = 0.1 ^1^	7.0 (7.0, 7.0) ^a^	7.0 (6.0, 7.0) ^a^	6.0 (5.0, 7.0) ^b^	5.0 (3.0, 7.0) ^c^	*p* < 0.0001
Proportion of items tracked on day of intake, Mdn (IQR) (range: 0 to 1)	0.74 (0.59, 0.90)	0.68 (0.57, 0.80)	*p* = 0.6	0.66 (0.57, 0.82)	0.75 (0.63, 0.85)	*p* = 0.2	0.89 (0.72, 0.99) ^a^	0.70 (0.56, 0.98) ^a,b^	0.74 (0.42, 0.83) ^b^	0.69 (0.41, 0.91) ^b^	*p* = 0.001
Number of logging sessions, Mdn (IQR)	22.5 (20.0, 33.0)	23.0 (16.0, 25.0)	*p* = 0.4	22.0 (20.0, 26.0)	24.0 (16.0, 30.0)	*p* = 0.7	7.0 (6.0, 9.0) ^a^	6.0 (4.0, 8.0) ^a,b^	5.0 (4.0, 7.0) ^b^	4.0 (3.0, 6.0) ^c^	*p* < 0.0001

^1^ Medium effect size ($\omega_{p}^{2}$ = 0.09). ^2^ Cells with different superscripts (a, b, c) indicate significant differences (*p* < 0.05) between weeks. Abbreviations: Mdn (IQR) = median (interquartile range).

**Table 3:** Scores for intrinsic motivation subdomains at follow-up.

Intrinsic Motivation Subscales (Range: 1 to 7)	Caregiver Praise			Gamification		
	No (n = 10)	Yes (n = 9)	Main Effect	No (n = 10)	Yes (n = 9)	Main Effect
Interest/enjoyment, Mdn (IQR)	5.3 (4.0, 5.9)	2.7 (2.1–5.4)	*p* = 0.4	2.6 (1.6–4.9)	5.4 (5.3–6.3)	*p* = 0.048
Perceived competence, Mdn (IQR)	6.1 (4.6, 6.8)	4.0 (1.8, 6.2)	*p* = 0.3	4.2 (3.2, 6.2)	6.2 (5.8, 6.8)	*p* = 0.06
Perceived choice, Mdn (IQR)	5.3 (4.6–6.2)	5.8 (4.8–6.4)	*p* = 0.3	5.5 (4.6–6.4)	5.8 (4.6–6.2)	*p* = 0.4
Pressure/Tension, Mdn (IQR)	1.5 (1.5–1.8)	2.5 (1.8–3.3)	*p* = 0.1	1.6 (1.5–2.5)	2.0 (1.5–3.5)	*p* = 0.2

Abbreviations: Mdn (IQR) = median (interquartile range).

## Data Availability

Due to the small sample size and the limited geographic area from which participants were recruited, there is an increased risk of deductive disclosure. To protect participant confidentiality, the dataset is not publicly available.

## Supplementary Materials

The following supporting information can be downloaded at: https://www.mdpi.com/article/10.3390/nu17213341/s1, File S1: Formative Study Methods and Results, Figure S1: Handout of virtual pet stages of evolution provided to children and caregivers for feedback, Table S1: Responses to usability and acceptability survey by condition.

## Abbreviations

The following abbreviations are used in this manuscript:

ANCOVA	Analysis of covariance
ANOVA	Analysis of variance
DSM	Dietary self-monitoring
IQR	Interquartile range
PR	Positive reinforcement
SD	Standard deviation
URL	Uniform resource locator
