# Supplementary material for: Enhancing Child Digital Dietary Self-Monitoring via Positive Reinforcement: Proof-of-Concept Trial
Source: Nutrients. 2025 Oct 24;17(21):3341. doi: 10.3390/nu17213341 (PMC12610852; doi:10.3390/nu17213341)
Supplement: Supplementary file 1 [file nutrients-17-03341-s001.zip › Figure S1.pdf]

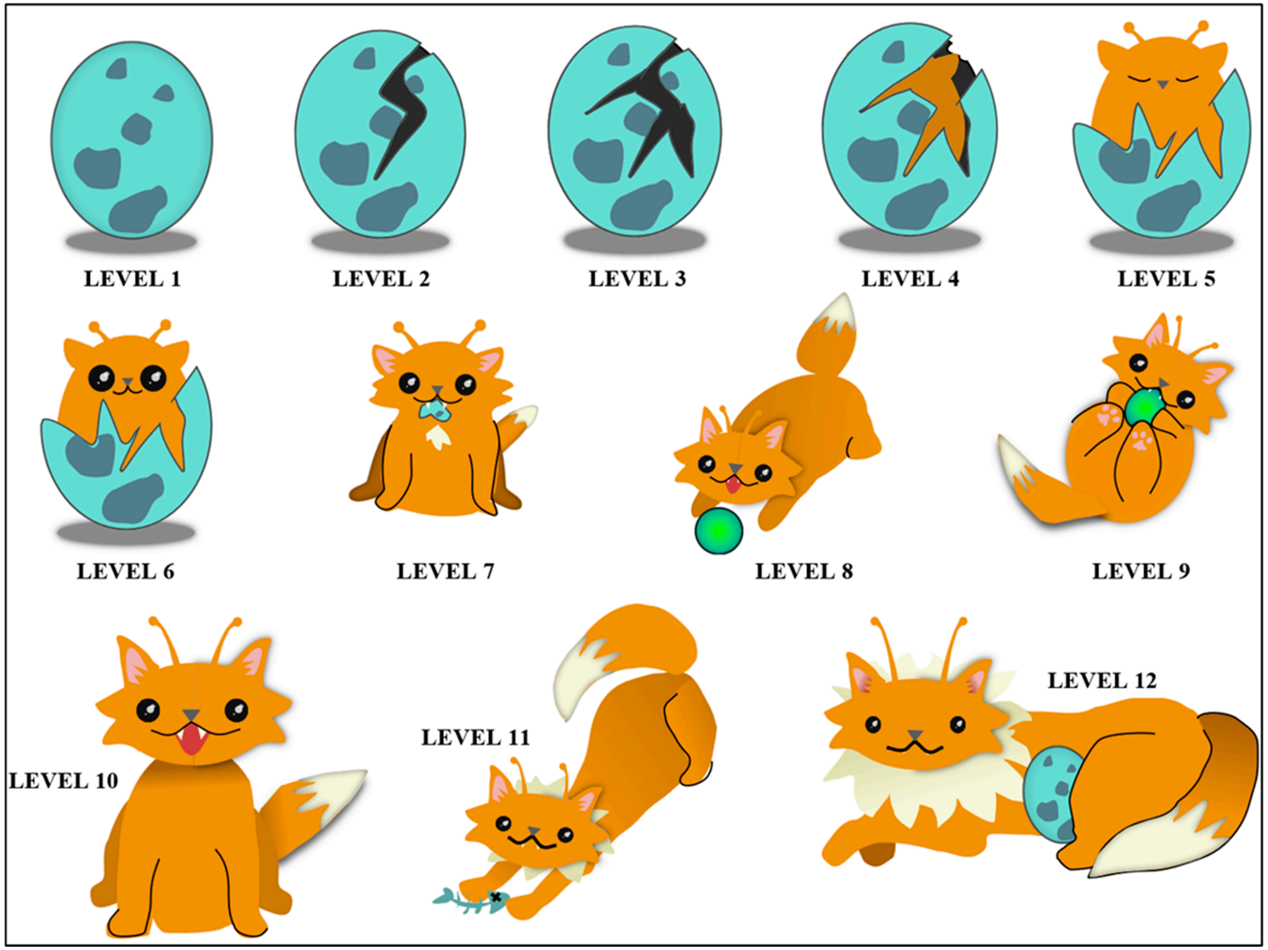

Figure S1. Handout of virtual pet stages of evolution provided to children and caregivers for feedback.
