# Supplementary material for: Enhancing Child Digital Dietary Self-Monitoring via Positive Reinforcement: Proof-of-Concept Trial
Source: Nutrients. 2025 Oct 24;17(21):3341. doi: 10.3390/nu17213341 (PMC12610852; doi:10.3390/nu17213341)
Supplement: Supplementary file 1 [file nutrients-17-03341-s001.zip › File S1.pdf]

Formative Study Methods and Results

Supplementary File S.1: Formative study methods

S.1.1 Study Design and Primary Outcomes

Formative procedures consisted of a single in-person usability and acceptability testing session. A laptop with a beta version of the web-based DSM log was used during the appointment. The log included four features: the ability to log foods and beverages, a "help me" feature (which described the food groups that would be targeted in the proof-of-concept trial, with a description of serving sizes), a button to mark logging complete for the day, and a caregiver check-in feature (which allowed caregivers to indicate they have reviewed their child's DSM for that day). Gamification was not yet integrated into the log during beta testing. Thus, a printed handout with virtual pet design along with a verbal description of how gamification would function in the final version of the log was provided for feedback. The primary outcomes were the usability and acceptability of the beta log as reported by children and their caregivers.

S.1.2 Participants

Recruitment occurred from August through November of 2023. Children aged 8-12 years, with an adult caregiver willing to participate, were eligible for the study. Child-caregiver dyads had to live in the greater Knoxville area and be able to communicate and read in English. Children had to be able to read at a third grade reading level or above, as reported by an adult caregiver, and were excluded if they had any major psychiatric disorders or organic brain syndromes. The study protocol was approved by the Institutional Review Board at the University of Tennessee, Knoxville (UTK IRB-22-07259-XP).

Families were recruited from a list of potential participants generated from the Families Becoming Healthy Together (FBHT) Program (PI: Hollie Raynor, PhD; UTK IRB-18-04910-XP), a family-based lifestyle intervention study conducted at UTK.

Study staff contacted potentially eligible families to complete a phone screening. During the phone screening, researchers provided an overview of the study and asked eligibility screening questions. Eligible families were invited to complete a single in-person session The session began with an orientation that included a detailed overview of the study, after which written informed consent and assent were collected.

S.1.3 Flow of Session

Two study staff were present during testing sessions, an interviewer and a second observer, took notes on nonverbal responses (e.g., body language or facial expressions that indicated emotions such as boredom, frustration, confusion). All testing sessions were audio recorded using Zoom with the camera off.

After consent and assent were collected, the interviewer provided an overview of the purpose of the DSM log and its basic features to both the child and caregiver. Participants were informed that their opinions and feelings about the log were important, whether positive or negative, and that there were no right or wrong responses. After this introduction, the caregiver was asked to wait in a separate room while the child tested the DSM log with researcher staff. Children were separated from caregivers to prevent caregivers from influencing the child's interaction with the log or making remarks that might alter the child's perception or experience. A concurrent think-aloud protocol was implemented with the child to help identify problems with design and acceptability. Think-aloud protocols have been shown to be effective for usability testing in children.138

First, children were provided with a standardized list of foods and beverages with amounts to practice logging in the DSM log. The logging feature allowed users to record food and drinks consumed using three fields: "food or drink description," "amount," and "servings."

In addition to thinking aloud, children were asked to mention anything they experienced that related to the following questions: 1) "Did you need help completing the task?" and 2) "What, if anything, happened that

you did not expect or want?"138 These questions were printed on a piece of paper and placed next to the child. The interviewer prompted the child to keep talking or to remember the questions if they fell silent.

Next, study staff asked children to identify what food group specific foods and beverages belonged to and what the appropriate serving size was by navigating through the "help me" feature. The help feature allowed users to look up serving sizes for items from food groups that would be targeted in the proof-of-concept trial (i.e., fruits, vegetables, sweet and salty snack foods, and sugar-sweetened beverages). For fruits, vegetables, and snack foods, help pages displayed tables listing commonly consumed foods and drinks with serving sizes in alphabetical order. Given that all sugar-sweetened beverages have the same serving size, the help page displayed a graphic of a bottle, can, and cup along with written text describing a serving as 8 fluid ounces. During help feature testing, the children were asked to keep the same two think-aloud questions in mind. The interviewer recorded which food groups and servings sizes the children identified correctly.

Finally, a printout of the virtual pet that would be included in the final version of the log showing all 12 levels of its evolution was provided for child feedback (Appendix B). After describing how the virtual pet would function in the DSM log (i.e., leveling up with points over time), research staff asked the child three questions: (1) What do you like about this pet? (2) What do you not like about this pet? and (3) Would you want to use the log more to help your pet grow over time?

Finally, the children were asked to complete a short usability and acceptability survey.139

After completing the survey, the caregivers were asked to return to the room. A parallel procedure (concurrent think-aloud activities and usability and acceptability survey) was conducted with adult caregivers. In addition to testing the logging and "help me" features and providing feedback on the virtual pet, caregivers were also asked to practice completing a caregiver check-in. Finally, the caregiver was asked to complete a short usability and acceptability survey like the one administered to the child.

At the completion of the session, the family received two $10 gift cards (one for the child and one for the caregiver).

S.1.4 Measures

Usability and acceptability survey. A survey was adapted from the usability and acceptability satisfaction questionnaire implemented in Marsac et al that asked about visual appeal, function, and comprehensibility.139 Though not validated, the original survey provided a framework for adapting simple, straightforward questions related to usability and acceptability appropriate for the formative testing phase.

S.1.5 Data Analysis

For qualitative analysis, the auto-transcription feature of Zoom was used to generate initial transcripts from the think-aloud portions of the session. All automated transcripts were reviewed for accuracy and revised as needed.

Two staff independently coded each transcript to identify comments related to the usability and acceptability using a priori codes (Table S1.1). These codes were provided to the second coder, along with a training video, prior to beginning the coding process. NVivo 14 (Lumivero, Denver, CO) was used to code transcripts and compare inter-coder responses. After independent review, all coding was combined into a single project in NVivo so that agreements and disagreements could be identified. The two coders then met to reconcile disagreements and finalize codes. Percent agreement was calculated as the total number of agreements divided by the total number of agreements plus disagreements. If a majority (3 of 5) of children or caregivers reported the same usability or acceptability issue, changes to this feature were made prior to the proof-of-concept trial.

For quantitative analysis, descriptive statistics were used to determine the percentage of correct responses provided during help feature testing. Additionally, frequency of responses for each item on the usability and acceptability satisfaction questionnaire was calculated.

Table S1.1. A priori codes and definitions for qualitative analysis of transcripts produced from think-aloud procedures.

Codes	Definitions
Usability	
Burden	Comments related to a feature being burdensome, e.g., taking too much time, being redundant, being "clunky"
Confusion and needing help	Comments related to a feature being confusing, i.e., the user is unsure how to proceed or needs help to use it
Ease of use	Comments related to a certain feature being easy to use
Limitations and suggestions	Comments related to things the user wishes were present, as well as suggestions for addressing the limitation
Acceptability	
Design and Appearance, Negative	Negative comments about the visual design or appearance (color scheme, layout, etc.); this does NOT include comments on functionality
Design and Appearance, Positive	Positive comments about the visual design or appearance (color scheme, layout, etc.); this does NOT include comments on functionality
Motivation	Comments related to a user's motivation to use the DSM log

Appendix S.2: Formative study results

S.2.1 Child and Caregiver Demographics

In total, 5 children and their caregivers participated in testing sessions. The sample included 2 female and 3 male children; all caregivers were female. The average age of child participants was 10.4 ± 1.5 years of age.

S.2.2 Qualitative Evaluation (Think-Aloud Procedure)

Usability: Burden

When testing the logging feature, both caregivers and children mentioned inefficient navigation between log features, such as needing extra clicks or having to navigate through unnecessary pages.

Example 1

Caregiver 1: *It would be helpful, I feel like, if it went back to instead of like home every time like it automatically went back to log a food or beverage, and then you have the option to click home, or maybe home or continue. That way you're not constantly having to go back in and go back to that home page.*

Example 2

Child 5, 12 years old: *Maybe, this is just my opinion here, but like with the log, button, it might be more convenient just to like immediate like after you log it just for to immediately come back to the [home page].*

Additionally, a couple of children made comments related to the time burden related to finding a food or beverage in the help feature.

Usability: Confusion and Needing Help

When testing the logging feature, caregivers expressed concern that children in the targeted age range might find the logging fields (i.e., item description, amount, and servings) confusing, specifically which information should be entered in which field. Furthermore, most of the children expressed that calculating or inputting the amount of food or beverage consumed might be challenging. In particular, families expressed that children in the targeted age range might feel confused about how to use units and how to figure out serving sizes. Almost all caregivers mentioned that children might struggle with entering fractions.

Example 1

Caregiver 3: *Like I said, if it were him, I think deciding which goes in which [input field], I think, he would, but I think you know, especially he's on the younger end of your range, so I think once he got used to it, he could do it. It would maybe take him a little bit of time.*

Example 2

Child 5, 12 years old: *So that was confusing too, like the ounce, and maybe like more distinguish between the amount and servings.*

In addition to the noted time burden related to the help feature, several comments indicated participant confusion and needing help. The help feature consisted of four webpages, one for each targeted food group. The fruit, vegetable, and snack food pages each included tables of common foods in alphabetical order along with their serving sizes. In contrast, the sugar-sweetened beverage page relied on a visual graphic illustrating what a serving size looks like for common containers (e.g., can of soda). The majority of both children and caregivers felt the inconsistency of presenting the graphic on the sugar-sweetened beverage help page versus the tables on the other help pages was confusing. Additionally, when testing the help feature, several children needed help finding a food or drink in the lists.

Example 1

Child 2, 10 years old: *The thing I have to say about this is the [sugar-sweetened beverage] pictures kind of confuse me a little.*

Example 2

Child 1, 11 years old: *Can't find the broccoli, finding the broccoli.*

Interviewer: *You need help finding the broccoli?*

Child 1: *Yes, I can't find the broccoli.*

Usability: Ease of Use

Despite expressing some confusion and needing help with logging, a majority of children and caregivers made comments related to the ease of using the logging feature.

Example 1

Interviewer: *Any part of this we feel like [Child] would need help with?*

Caregiver 2: *No, I think she would get it.*

Example 2

Child 5, 12 years old: *Yeah, after a couple of times I think people will get the hang of doing it. It just takes a while to learn like anything.*

Usability: Limitations and Suggestions

Additionally, four caregivers suggested features to customize the pet based on the children's preferences.

Example 1

Caregiver 2: *Do we have any choices of other animals? Could they be a dinosaur if it's a guy or can it, if you give it multiple animals like, if you want to grow butterfly, or you know not just a cat.*

Example 2

Caregiver 5: *Yeah, like, I mean, there could be different pets. But there could be like maybe little things that I could add to my pet or feed my pet, you know. Take care of my pet, trim my pet. Things like that might be cool.*

Acceptability: Design and Appearance, Positive

Most children made positive comments related to the general design and appearance of the virtual pet and its different stages of evolution. Several participants compared the design to other popular character they already liked.

Example 1

Caregiver 1: *So this one where it involves a pet that you can kind of, you know, watch develop, I think that's more universal.*

Example 2

Child 1, 11 years old: *It's a cat. Adorable.*

Acceptability: Design and Appearance, Negative

No negative comments were made about any singular feature of the design or appearance by a majority of children or caregivers.

Acceptability: Motivation

When asked whether they/their child would want to use the log more to help the pet grow over time, all children and caregivers responded affirmatively.

Example 1

Caregiver 1: *I like that it starts as an egg and goes all the way up to like a full-grown pet. Like I feel like that's gonna motivate the child to go on there, log their foods, because they're gonna want to see their pet grow. It reminds me a lot of like, you know, when I was younger Tamagotchis and trying to keep your little Tamagotchi alive. I like, I like that it's very Pokémon-like, because I feel like that's gonna appeal to a lot of kids. But then, on the other end of it, it's very just, it's cute, which, if so, if a child, maybe not into Pokémon, because it's such a cute little pet I think that that's gonna appeal to the other kids.*

Example 2

Caregiver 3: *I like that it grows and it's a slow growth, so that they're more apt to log to get into out here.*

S.2.3 Quantitative Evaluation (Questionnaires)

Help Feature Questionnaire

During the think-aloud procedure, participants were asked by the interviewer to use the help feature to identify the food group and servings sizes of 8 pre-selected food and beverage items. Children identified the correct food group 100% of the time and the correct serving size 87.5% of the time. Caregivers identified the correct food group 100% of the time and the correct serving size 97.5% of the time. This suggests that both child and caregivers were able to use the help feature correctly to a high degree.

Usability and Acceptability Satisfaction Questionnaire

Results for the usability and acceptability survey are presented in **Table S1.2**. In general, children and caregivers responded positively to log design, appearance, and function. Consistent with comments made during the think-aloud procedures, a majority of children rated the log as "somewhat" rather than "very" easy to use, while all caregivers rated it as "very easy" to use. Furthermore, two children and two caregivers rated the help feature as "somewhat confusing," which was reflected in comments made related to the sugar-sweetened beverage graphic, as well as burden related to finding foods/beverages in the help feature tables.

Table S1.2. This is a table caption.

Survey Item	Child n (%)	Parent n (%)
The tool is easy to use		
Very easy	2 (40%)	5 (100%)
Somewhat easy	3 (60%)	0 (0%)
Not at all easy	0 (0%)	0 (0%)
I would use the tool again		
Yes	4 (80%)	5 (100%)
No	0 (0%)	0 (0%)
Maybe / I'm not sure	1 (20%)	0 (0%)
Logging foods and drinks is confusing		
Very confusing	0 (0%)	0 (0%)
Somewhat confusing	3 (60%)	2 (40%)
Not at all confusing	2 (40%)	3 (60%)
Using the "help me" feature is confusing		
Very confusing	0 (0%)	0 (0%)
Somewhat confusing	2 (40%)	2 (40%)
Not at all confusing	3 (60%)	3 (60%)
The "help me" feature had too many words		
Yes	2 (40%)	0 (0%)
No	1 (20%)	5 (100%)
Maybe / I'm not sure	2 (40%)	0 (0%)
Using the caregiver check-in feature is confusing		
Very confusing	NA	0 (0%)
Somewhat confusing	NA	0 (0%)
Not at all confusing	NA	5 (100%)
The tool is fun to use / My child would enjoy using this tool		
A lot	2 (40%)	3 (60%)
Somewhat	3 (60%)	2 (40%)
Not at all	0 (0%)	0 (0%)
I like the appearance of the tool		
A lot	3 (60%)	3 (60%)
Somewhat	2 (40%)	2 (40%)
Not at all	0 (0%)	0 (0%)
I like the things the tool can do		
A lot	4 (80%)	5 (100%)
Somewhat	1 (20%)	0 (0%)
Not at all	0 (0%)	0 (0%)
I like / my child would like the virtual pet the tool will use		
A lot	5 (100%)	4 (80%)
Somewhat	0 (0%)	1 (20%)
Not at all	0 (0%)	0 (0%)
I would recommend this too to other kids / families		
Yes	4 (80%)	5 (100%)
No	0 (0%)	0 (0%)
Maybe / I'm not sure	1 (20%)	0 (0%)

S.2.4 Improvements Prior to Proof-of-Concept Trial

Some areas for improvement were identified. First, navigation between features in the beta log was generally deemed cumbersome and burdensome. To address the burden related to clunky navigation, additional navigation buttons were added to the log prior to the proof-of-concept trial to ensure users could more quickly access the pages and features they desired.

One issue identified with the help feature was the inconsistency of the presentation of information between the sugar-sweetened beverage page (graphic) and the help pages for the other food groups (serving size table). All help pages were changed to a serving size table for consistency. Additionally, to address the burden of time and the confusion related to finding items on the help pages, families in the proof-of-concept trial were shown how to use keyword search to look for specific items.

Multiple families also expressed concerns that children in the targeted age range might struggle to understand, determine, and input the correct item descriptions, amounts, and servings for consumed foods/beverages. To address these challenges in the proof-of-concept trial, more intensive training (including in-depth instructions for each field and a practice session with research staff) was provided to families at their baseline appointment. Additionally, caregivers were encouraged to answer any questions their children had during the active DSM period.

While the virtual pet was largely praised, a majority of caregivers also suggested enhancements that would allow options to customize or interact with the virtual. However, the design of sophisticated customization capabilities was outside the scope of the current project. No other areas were identified for improvement for the virtual pet feature design or log function.
