# Supplementary material for: Enhancing Child Digital Dietary Self-Monitoring via Positive Reinforcement: Proof-of-Concept Trial
Source: Nutrients. 2025 Oct 24;17(21):3341. doi: 10.3390/nu17213341 (PMC12610852; doi:10.3390/nu17213341)
Supplement: Supplementary file 1 [file nutrients-17-03341-s001.zip › Table S1.pdf]

Table S1. Responses to usability and acceptability survey by condition.

Survey Item	BASIC (n=5) Child / Caregiver	PRAISE (n=5) Child / Caregiver	GAME (n=5) Child / Caregiver	GAME+PRAISE (n=4) Child / Caregiver
The log was easy to use.				
Very easy	5 (100%) / 0 (0%)	3 (60%) / 2 (40%)	5 (100%) / 5 (100%)	2 (50%) / 3 (75%)
Somewhat easy	0 (0%) / 5 (100%)	2 (40%) / 3 (60%)	0 (0%) / 0 (0%)	2 (50%) / 1 (25%)
Not at all easy	0 (0%) / 0 (0%)	0 (0%) / 0 (0%)	0 (0%) / 0 (0%)	0 (0%) / 0 (0%)
Logging foods and drinks was confusing.				
Very confusing	0 (0%) / 0 (0%)	2 (40%) / 0 (0%)	0 (0%) / 0 (0%)	1 (25%) / 0 (0%)
Somewhat confusing	2 (40%) / 0 (0%)	2 (40%) / 2 (40%)	0 (0%) / 0 (0%)	2 (50%) / 1 (25%)
Not at all confusing	3 (60%) / 5 (100%)	1 (20%) / 3 (60%)	5 (100%) / 5 (100%)	1 (25%) / 3 (75%)
Using the "help me" feature was confusing.				
Very confusing	0 (0%) / 0 (0%)	1 (20%) / 0 (0%)	0 (0%) / 0 (0%)	1 (25%) / 0 (0%)
Somewhat confusing	2 (40%) / 0 (0%)	0 (0%) / 0 (0%)	0 (0%) / 0 (0%)	1 (25%) / 0 (0%)
Not at all confusing	3 (60%) / 5 (100%)	4 (80%) / 5 (100%)	5 (100%) / 5 (100%)	2 (50%) / 4 (100%)
The "help me" feature had too many words.				
Yes	1 (20%) / 0 (0%)	0 (0%) / 0 (0%)	0 (0%) / 0 (0%)	1 (25%) / 0 (0%)
No	2 (40%) / 3 (60%)	3 (60%) / 3 (60%)	4 (80%) / 4 (80%)	1 (25%) / 2 (50%)
Maybe / I'm not sure	2 (40%) / 2 (40%)	2 (40%) / 2 (40%)	1 (20%) / 1 (20%)	2 (50%) / 2 (50%)
Using the caregiver check-in feature was confusing.[1]				
Very confusing	NA / 0 (0%)	NA / 0 (0%)	NA / 0 (0%)	NA / 0 (0%)
Somewhat confusing	NA / 1 (20%)	NA / 0 (0%)	NA / 2 (40%)	NA / 0 (0%)
Not at all confusing	NA / 4 (80%)	NA / 5 (100%)	NA / 3 (60%)	NA / 4 (100%)
The log was fun to use.[2]				
A lot of fun	1 (20%) / NA	1 (20%) / NA	3 (60%) / NA	2 (50%) / NA
Somewhat fun	2 (40%) / NA	2 (40%) / NA	2 (40%) / NA	1 (25%) / NA
Not at all fun	2 (40%) / NA	2 (40%) / NA	0 (0%) / NA	1 (25%) / NA
My child enjoyed using this log.[1]				
A lot	NA / 0 (0%)	NA / 1 (20%)	NA / 1 (20%)	NA / 2 (50%)
Somewhat	NA / 3 (60%)	NA / 1 (20%)	NA / 4 (80%)	NA / 1 (25%)
Not at all	NA / 2 (40%)	NA / 3 (60%)	NA / 0 (0%)	NA / 1 (25%)
I liked the appearance of the log.				
A lot	2 (40%) / 0 (0%)	1 (20%) / 0 (0%)	3 (60%) / 2 (40%)	3 (75%) / 2 (50%)
Somewhat	3 (60%) / 4 (80%)	1 (20%) / 3 (60%)	2 (40%) / 3 (60%)	1(25%) / 2 (50%)
Not at all	0 (0%) / 1 (20%)	3 (60%) / 2 (40%)	0 (0%) / 0 (0%)	0 (0%) / 0 (0%)
I would recommend this log to other kids / families.				

Yes	1 (20%) / 1 (20%)	2 (40%) / 1 (20%)	5 (100%) / 3 (60%)	1(25%) / 2 (50%)
No	1 (20%) / 1 (20%)	1 (20%) / 0 (0%)	0 (0%) / 0 (0%)	1 (25%) / 0 (0%)
Maybe / I'm not sure	3 (60%) / 3 (60%)	2 (40%) / 4 (80%)	0 (0%) / 2 (0%)	2 (50%) / 2 (50%)
I would use the log again.				
Yes	1 (20%) / 1 (20%)	2 (40%) / 1 (20%)	2 (40%) / 3 (60%)	3 (75%) / 2 (50%)
No	0 (0%) / 1 (20%)	2 (40%) / 1 (20%)	0 (0%) / 0 (0%)	1 (25%) / 0 (0%)
Maybe / I'm not sure	4 (80%) / 3 (60%)	1 (20%) / 3 (60%)	3 (60%) / 2 (40%)	0 (0%) / 2 (50%)
The log was easy to use.				
Very easy	5 (100%) / 0 (0%)	3 (60%) / 2 (40%)	5 (100%) / 5 (100%)	2 (50%) / 3 (75%)
Somewhat easy	0 (0%) / 5 (100%)	2 (40%) / 3 (60%)	0 (0%) / 0 (0%)	2 (50%) / 1 (25%)
Not at all easy	0 (0%) / 0 (0%)	0 (0%) / 0 (0%)	0 (0%) / 0 (0%)	0 (0%) / 0 (0%)
